# A Novel Newborn Rat Kernicterus Model Created by Injecting a Bilirubin Solution into the Cisterna Magna

**DOI:** 10.1371/journal.pone.0096171

**Published:** 2014-05-05

**Authors:** Sijie Song, Ying Hu, Xianfang Gu, Feifei Si, Ziyu Hua

**Affiliations:** 1 Department of Neonatology, Children's Hospital of Chongqing Medical University, Chongqing, China; 2 Ministry of Education Key Laboratory of Child Development and Disorders, Chongqing Medical University, Chongqing, China; 3 Chongqing Key Laboratory of Translational Medical Research in Cognitive Development and Learning and Memory Disorders, Chongqing Medical University, Chongqing, China; 4 Key Laboratory of Pediatrics in Chongqing, Chongqing Medical University, Chongqing, China; 5 Chongqing International Science and Technology Cooperation Center for Child Development and Disorders, Children's Hospital, Chongqing Medical University, Chongqing, China; 6 Department of Pediatrics, Southwest Hospital, Third Military Medical University, Chongqing, China; 7 Department of Neonatology, Children's Hospital of Kaifeng, Henan, China; St Michael's Hospital, University of Toronto, Canada

## Abstract

**Background:**

Kernicterus still occurs around the world; however, the mechanism of bilirubin neurotoxicity remains unclear, and effective treatment strategies are lacking. To solve these problems, several kernicterus (or acute bilirubin encephalopathy) animal models have been established, but these models are difficult and expensive. Therefore, the present study was performed to establish a novel kernicterus model that is simple and affordable by injecting unconjugated bilirubin solution into the cisterna magna (CM) of ordinary newborn Sprague-Dawley (SD) rats.

**Methods:**

On postnatal day 5, SD rat pups were randomly divided into bilirubin and control groups. Then, either bilirubin solution or ddH_2_O (pH = 8.5) was injected into the CM at 10 µg/g (bodyweight). For model characterization, neurobehavioral outcomes were observed, mortality was calculated, and bodyweight was recorded after bilirubin injection and weaning. Apoptosis in the hippocampus was detected by H&E staining, TUNEL, flow cytometry and Western blotting. When the rats were 28 days old, learning and memory ability were evaluated using the Morris water maze test.

**Results:**

The bilirubin-treated rats showed apparently abnormal neurological manifestations, such as clenched fists, opisthotonos and torsion spasms. Bodyweight gain in the bilirubin-treated rats was significantly lower than that in the controls (*P*<0.001). The early and late mortality of the bilirubin-treated rats were both dramatically higher than those of the controls (*P* = 0.004 and 0.017, respectively). Apoptosis and necrosis in the hippocampal nerve cells in the bilirubin-treated rats were observed. The bilirubin-treated rats performed worse than the controls on the Morris water maze test.

**Conclusion:**

By injecting bilirubin into the CM, we successfully created a new kernicterus model using ordinary SD rats; the model mimics both the acute clinical manifestations and the chronic sequelae. In particular, CM injection is easy to perform; thus, more stable models for follow-up study are available.

## Introduction

Hyperbilirubinemia is a common phenomenon among neonates. Severe hyperbilirubinemia may cause kernicterus, which may lead to death or lifelong neurological impairments, such as motor-development delay, sensorineural hearing loss, epilepsy, cerebral palsy and mental retardation [1]. With advancements in perinatal medicine, the incidence of kernicterus has been reduced dramatically; however, kernicterus still occurs in developed countries in which perinatal healthcare is well developed, and the incidence is even higher in developing countries [2]–[5]. The most common outcomes of kernicterus are death and survival with nervous system sequelae due to the lack of specific therapeutic strategies [1]; therefore, there is an urgent need for the development of other effective treatments.

Unfortunately, to date, the mechanisms underlying bilirubin neurotoxicity are incompletely understood. In an effort to develop effective strategies, intensive research has been conducted over the last few decades. In 1938, Gunn first found a new mutation leading to jaundice in rats and named the strain the Gunn rat [6]. To establish an acute bilirubin encephalopathy (ABE) animal model, sulfadimethoxine (a displacer of unconjugated bilirubin from albumin) and phenylhydrazine (an inducer of hemolysis) have been used in Gunn rats [7]–[11]. However, models using Gunn rats are limited, as maximal hyperbilirubinemia occurs on postnatal day 16 (P16) [10], instead of the particularly vulnerable first week in human newborns. Second, the levels of unconjugated bilirubin (UCB), the most critical risk factor for bilirubin encephalopathy, are not significantly higher than those in controls, which is partly due to the extrahepatic conjugation that occurs in this rat model [11]–[13]. Moreover, the drugs used to induce hyperbilirubinemia might impact the brain directly or indirectly [8]–[11]. Recently, Nghia Nguyen and Ryoichi Fujiwara created gene knockout hyperbilirubinemia mice (*Ugt1*-null mouse and the humanized *Ugt-1* mouse) [14]–[15]. However, all *Ugt1*-null mice die within 11 days, and no studies have examined the impact of bilirubin neurotoxicity in these mice. In addition, only a small number of humanized *Ugt-1* mice (10%) show central nervous system (CNS) toxicity, seizures and death at P7–P14, and therefore, sufficient kernicterus models are not available for acute studies or studies on chronic sequelae. Furthermore, these gene knockout mouse models might be difficult and expensive. Therefore, a simple and affordable animal model of kernicterus is required to closely mimic the clinical manifestations of ABE in human newborns and to directly study the molecular biological mechanisms of kernicterus; such a model would be suitable for exploring effective strategies to intervene in bilirubin neurotoxicity.

In several studies focusing on CNS diseases, cisterna magna (CM) puncture has been widely used to inject drugs or to collect cerebrospinal fluid (CSF) [16]–[19]. In addition, it is reported that CSF is a transport conduit for substance exchange (e.g. neurotransmitters, neurohormones) between the CSF and brain tissue. In addition, the mechanism underlying the penetration of substances contained in the CSF into the brain from the CM is complex, with preferential entry along the ventral side of the brain, particularly into the hypothalamus and brainstem, which are severely damaged in kernicterus [9], [11], [20]. Therefore, we hypothesized that UCB would also distribute throughout the brain after injection into the CM.

In the present study, our objective was to establish a new kernicterus model using newborn Sprague-Dawley (SD) rats by injecting UCB into the CM. This is the first study to successfully generate a new kernicterus model using ordinary SD rats by injecting bilirubin into the CM; this model shows not only the acute clinical manifestations but also the chronic sequelae of bilirubin encephalopathy. Therefore, it is possible to more easily and directly study bilirubin neurotoxicity *in vivo* and to achieve stable models in large numbers.

## Materials and Methods

### 1 Model establishment

#### 1.1 Ethics Statement and Animals

All animal studies were performed in accordance with the Guide for the Care and Use of Laboratory Animals of the National Institutes of Health, and all efforts were made to minimize suffering. The Ethics Committee of the Children's Hospital of Chongqing Medical University (Permit Number: SYXK2007-0016) approved all experiments. All animals (SPF grade) were purchased from the Animal Experiment Center of Chongqing Medical University. All animals were kept in standard laboratory housing with a 12∶12 hour light:dark cycles and a constant temperature (23±2°C).

#### 1.2 Bilirubin solution preparation and model establishment procedures

Bilirubin solution preparation was as follows: bilirubin (Sigma-Aldrich, USA) was dissolved in 0.5 M NaOH solution (100 mg/ml) and diluted in ddH_2_O to a concentration of 10 mg/ml, and the pH was adjusted to 8.5 with HCl (0.5 M); the solution was stored at −20°C, in the dark [21]. Preliminary testing indicated that a dose of 10 µg/g (bodyweight) bilirubin produces characteristic neurological manifestations in addition to a higher survival rate (Figure S1); accordingly, that dosage was used in our subsequent experiments. On postnatal day 5, SD rat pups (10–15 g) from the same litter were randomly divided into two groups: the bilirubin group and the control group (n = 8 at least in each group for each assay). Before model establishment, rat pups were anesthetized under diethyl ether; then, approximately 10–15 µl of CSF was released from the CM with a microsyringe to prevent intracranial hypertension. Bilirubin solution or ddH_2_O (pH = 8.5) was then injected into the CM at 10 µg/g (bodyweight) using a microsyringe (measuring range: 25 µl).

### 2 Animal model identification

#### 2.1 General conditions and neurobehavioral changes

Neurobehavioral changes were observed at 0–6/9–12/21–24 hours after model establishment. Autopsy (10%) was performed to determine whether the cerebrum and cerebellum were stained yellow without tissue injury or bleeding. Bodyweight was recorded daily for three consecutive days after initiation of the model and weaning (at P23) (PMD: post-modeling day; PWD: post-weaning day).

#### 2.2 Early and late mortality

After model establishment, the number of rat deaths in each group was recorded for mortality calculation: early mortality, death before PMD-7; late mortality, death between PMD-7 and postnatal day 28.

#### 2.3 Histology and flow cytometry (FCM)

According to preliminary testing (data not shown), rats were sacrificed at 24 hours after model establishment, and the cerebrum was immediately fixed with 4% paraformaldehyde, followed by paraffin embedding. Coronal paraffin sections (5 µm) were cut from the bregma optic chiasma and stained with Hematoxylin-Eosin (H&E). Morphological changes in the hippocampus were observed using light microscopy (Nikon, Japan).

The abovementioned paraffin-embedded coronal sections were used to observe apoptosis in the hippocampal neurons using the Terminal-deoxynucleotidyl Transferase Mediated Nick End Labeling (TUNEL) method. Additionally, the hippocampus was isolated, and the cells were collected with precooled PBS [22]; then, Annexin V-fluorescein isothiocyanate (FITC) and propidium iodide (PI) were added, mixed well and incubated for 15 min at room temperature, followed by FCM for apoptosis and necrosis detection [23].

#### 2.4 Morris water maze

Learning and memory ability were tested when the rats were 28 days old using the Morris water maze test, as described previously [24]–[25]. Briefly, the water maze consisted of a circular pool filled with water containing black ink (25±1°C) to a depth of 30 cm. An automatic tracking system (SLY-Water Maze System 2.0, China) was used to record escape latency and the swimming path. Each rat was tested for 8 days, with 4 trials per day from days 1-7 and 1 trial on day 8. Training was performed on days 1–3, the place navigation ability test was performed on days 4–7, and spatial probe ability was assessed on day 8. If the rat failed to find the safety platform within 60 seconds, the rat was guided onto the platform with a stick and forced to stay there for 30 seconds; in these cases, escape latency was recorded as 60 seconds. The platform was removed from the pool on day 8, and the rats were allowed to swim freely for 120 seconds as a probe trial. The number of times each rat crossed over the exact location of the platform and the percentage of time spent searching in the safety quadrant within 120 seconds were recorded.

#### 2.5 Western blotting for apoptosis detection

The hippocampus was separated 24 hours after model establishment, and cytosolic/mitochondrial proteins were extracted (KeyGEN BioTECH, China) and quantified using the BCA assay (Beyotime Biotechnology, China). Proteins were separated by 10% SDS-PAGE (Beyotime Biotechnology, China) and transferred to PVDF membranes (Bio-Rad, USA), which were then blocked with 5% non-fat milk at room temperature for 1 hour and incubated with rabbit-anti-Bcl-2, rabbit-anti-cleaved caspase-3 (both Cell Signaling, USA), rabbit-anti-Bax, rabbit-anti-Bcl-xL, rabbit-anti-cytochrome-c, rabbit-anti-COX-4 (all Epitomics, USA), and β-actin (4A Biotech, China) antibodies overnight at 4°C, followed by an HRP-conjugated secondary antibody for 1 hour; the membranes were developed using a super ECL assay kit (KeyGEN BioTECH, China) and a G-BOX imaging system (Syngene, UK).

### 3 Statistical analysis

Data are presented as the mean ± standard deviation. A statistical analysis was first performed with a normality test and a homogeneity test for variance. If the data were in compliance with a normal distribution and homogeneity of variance, Student's t test for independent samples was performed; otherwise, a rank sum test was used. Categorical data were analyzed using the Chi-square test. *P*<0.05 was considered to be statistically significant. All the statistical analyses were performed with SPSS software 17.0.

## Results

### 1 Neurological manifestation, general condition and mortality rate

#### 1.1 Neurological manifestations

**Figure 1:**
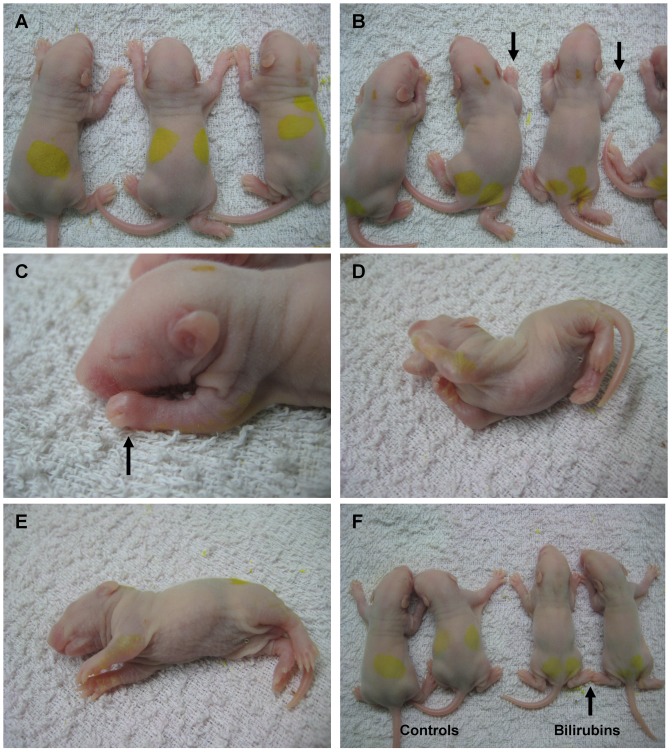
Clinical manifestations within 0.5–1 hour after bilirubin injection. (A) Control group rats. No abnormal neurobehavior was detected in the controls. (B)–(E) Representative images of abnormal neurobehavior in bilirubin-treated rats. Almost all of the bilirubin-treated pups showed clenched fists (B–C) (black arrow) and opisthotonos (D); some rats showed the latericumbent position (E). (F) Comparison of prostration in the two groups. The bilirubin-treated rats show decreased muscular tone (black arrow) and prostration.

Neurological manifestations, such as clenched fists, opisthotonos and torsion spasms were observed in all bilirubin-treated rats (n = 50) within 0.5–1 hour after bilirubin injection and persisted for 6–12 hours; in addition, some rats exhibited latericumbent positioning, rolling and limb fibrillation. No abnormal neurological features were observed in the controls (n = 46) (Fig 1 and Movie S1). Autopsy (10%) showed that the cerebrum, pons and cerebellum were stained yellow in the bilirubin-treated rats, and no operation-associated brain injury or bleeding was observed in either group (Figure S2).

#### 1.2 General condition and mortality rate

**Figure 2:**
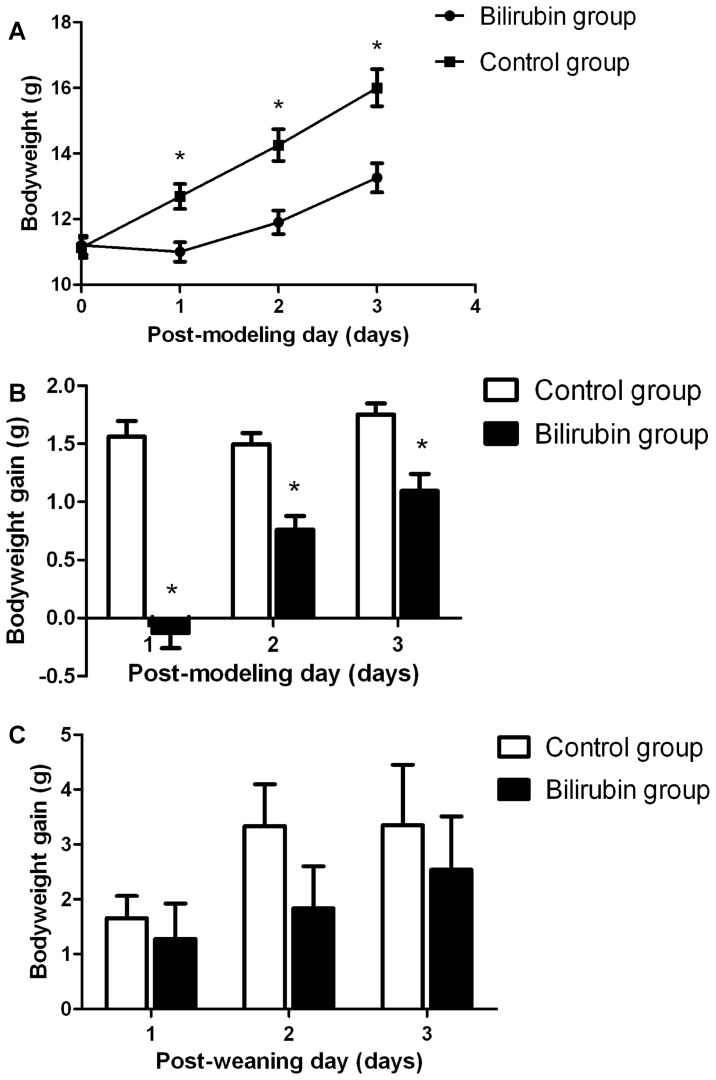
Bodyweight changes within 3 days after bilirubin injection or weaning. (A) Bodyweights of the bilirubin-treated rats and control rats on PMDs-1/2/3. (B) Bodyweight gains of the bilirubin-treated rats and control rats on PMDs-1/2/3. (C) Bodyweight gains of the bilirubin-treated rats and control rats on PWDs-1/2/3. (n = 12 in each group). *: *P*<0.01 vs control group. (PMD: post-modeling day; PWD: post-weaning day).

The rats in the bilirubin group showed a bodyweight loss on PMD-1, whereas the rats in the control group showed increased bodyweight (*P*<0.001) (Figs 2A and 2B). The bodyweight gains of the bilirubin group were somewhat improved but were still lower than those of the control group on PMD-2 (*P*<0.001) and PMD-3 (*P*<0.001) (Figs 2A and 2B). Although the bodyweight gains on PWD-1/2/3 in the bilirubin-treated rats were lower than those of the controls, no significant differences were observed between the two groups (Fig 2C).

As some rats were sacrificed for histological assays, there were 42 bilirubin-treated rats and 38 control rats remaining for mortality analysis. In the bilirubin group, 8 rats died before PMD-7, and 6 rats died after PMD-7 but before postnatal day 28; no control rats died before postnatal day 28. Thus, the early and late mortality rates of the bilirubin group rats were 19.05% and 14.29%, respectively, which were dramatically higher than those of the control group (*P* = 0.004 and 0.017, respectively).

### 2 Histology and FCM

**Figure 3:**
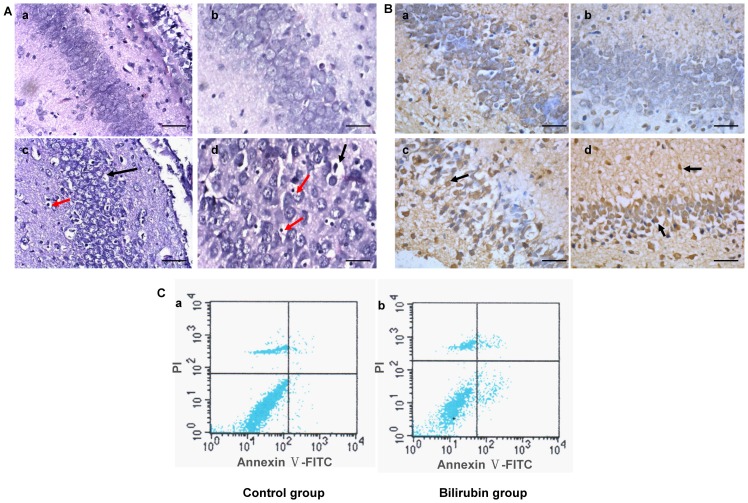
Histology and flow cytometry (FCM) at 24 hours after bilirubin injection. (A) H&E staining under light microscopy. Hippocampal nerve cells in the CA-3 region were morphologically normal in the control group (a, b). Cellular edema and swelling (c, d) (black arrow), and cell necrosis, including cytoplasmic condensation, endolysis, nuclear pyknosis, karyorrhexis and karyolysis (c, d) (red arrow), were observed in the bilirubin group (n = 8 in each group). (a), (c): 200×, scale bars = 200 µm; (b), (d): 400×, scale bars = 100 µm. (B) TUNEL assay under light microscopy. Few apoptotic cells in the CA-3 (a) and CA-1 (b) regions were observed in the controls. Apoptotic cells were observed in the CA-3 (c) and CA-1 (d) (black arrow) regions of the hippocampus in the bilirubin-treated rats. (n = 8 in each group). Scale bars = 100 µm. (C) Annexin V-FITC/PI (FCM) was used to determine the apoptotic (right lower quadrant) and necrotic (right upper quadrant) rates of the two groups. The apoptotic and necrotic rates of the controls were 1.33±0.36% and 1.26±0.89%, respectively (a). In the bilirubin group, the apoptotic and necrotic rates were 9.41±0.88% and 4.74±0.60%, respectively (b). There were significant differences in both the apoptotic and necrotic rates between the two groups (*P*<0.01). (n = 8 in each group).

H&E staining showed that hippocampal nerve cells in the CA-3 region were morphologically normal in the control group; however, cell necrosis (cytoplasmic condensation and endolysis, nuclear pyknosis, karyorrhexis and karyolysis) was observed in the bilirubin group (Fig 3A). Moreover, TUNEL staining showed obvious hippocampal nerve cell apoptosis in the CA-3 and CA-1 regions in the bilirubin-treated rats (Fig 3B), whereas apoptotic cells were rarely observed in the controls. Meanwhile, Annexin V-FITC/PI (FCM) showed both the apoptosis and necrosis of hippocampal nerve cells in the bilirubin group (Fig 3C); few apoptotic and necrotic cells were detected in the controls.

### 3 Morris water maze

**Figure 4:**
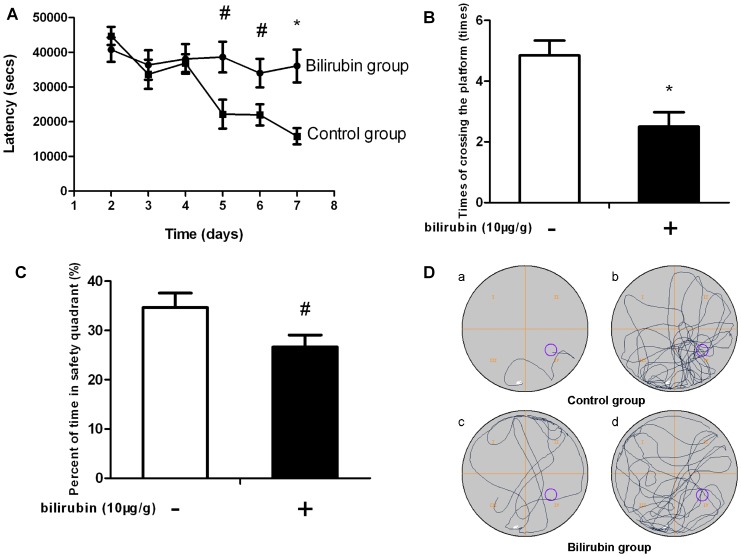
Learning and memory ability based on the Morris water maze test. (A) Escape latency to find the platform from days 2 to 7 of testing. Compared to controls, the bilirubin-treated rats showed a longer latency to escape onto the platform on days 5 to 7 (navigation trial). (B) The number of times the rats crossed over the platform location on day 8 (probe trial). The number of times that the bilirubin-treated rats crossed over the platform location was significantly lower when compared with that of the controls. (C) The percentage of time spent in the safety quadrant during the probe trial. Compared to controls, the bilirubin-treated rats spent less time in the platform safety quadrant. (D) Representative swimming paths. (a), (c): during the navigation trial on day 7. (b), (d): during the probe trial. Data are expressed as the mean ± SD (n = 12 in each group). *: *P*<0.01 vs control group; #: *P*<0.05 vs control group.

The escape latency in the control group shortened gradually. In contrast, the latency in the bilirubin-treated rats was dramatically longer than that of controls from days 5 to 7 (*P* = 0.001, 0.013 and 0.026, respectively), and it was only marginally decreased on days 6–7 (Fig 4A). The number of times that the bilirubin group rats crossed the platform location on day 8 (probe trial) was, on average, 2.50±1.51 (times), which was distinctly less than that in the control group (4.85±1.77 times) (*P* = 0.003) (Fig 4B). Additionally, the bilirubin group rats spent an average of 25.52±7.47% of 120 min in the safety quadrant containing the platform, which was significantly less when compared with the controls, at 34.66±10.54% (*P* = 0.03) (Fig 4C).

### 4 Effects of bilirubin on apoptosis-related molecules

**Figure 5:**
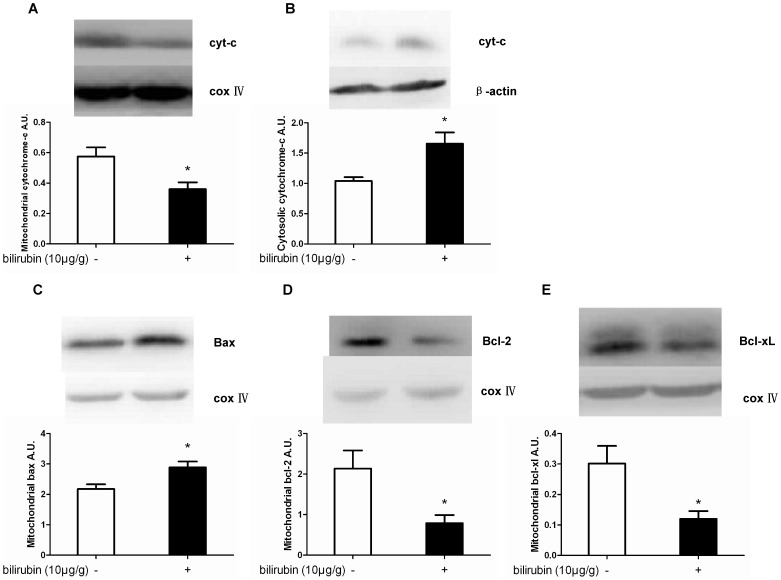
Effects of bilirubin on apoptosis-related molecules in the hippocampus at 24 hours after bilirubin injection. At 24 h after bilirubin injection, mitochondrial cytochrome-c (A) levels were significantly decreased compared with the control group; simultaneously, the cytosolic cytochrome-c (B) levels were significantly increased compared with the controls. Additionally, a significant increase of Bax in mitochondria (C) was observed. Meanwhile, Bcl-2 and Bcl-xL (D, E) expression in the mitochondria was dramatically decreased compared with the controls. (n = 12 in each group). *: *P*<0.01 vs the control group.

Our data revealed that bilirubin induced a significant decrease in mitochondrial cytochrome-c compared with the control group at 24 hours after the insult (*P* = 0.026), which is consistent with the finding that cytosolic cytochrome-c was simultaneously significantly increased compared with the controls (*P* = 0.009) (Figs 5A and 5B).

Furthermore, the expression levels of Bax, the proapoptotic protein of the Bcl-2 family, and Bcl-2 and Bcl-xL, which are antiapoptotic proteins, were examined. Compared with the control group, a significant increase in Bax expression in the mitochondria was observed in the bilirubin group at 24 h after bilirubin injection (*P*<0.001) (Fig 5C). Simultaneously, Bcl-2 and Bcl-xL expression in the mitochondria was dramatically decreased at 24 h in comparison with the controls (*P* = 0.036 and 0.007, respectively) (Figs 5D and 5E).

## Discussion

CM puncture, as a means of delivering drugs and chemotherapeutic agents, is used in clinical practice for sampling/removing CSF in cases of hydrocephalus, and its use in animals has also been widely described [16]–[19]. We successfully developed a convenient and typical kernicterus rat model by injecting a bilirubin solution into the CM. To the best of our knowledge, this is the first kernicterus rat model of this type that has been established, and ordinary SD rats can be used instead of Gunn rats, *Ugt1*-null mice, or humanized *Ugt-1* mice. The bilirubin group rats showed characteristic neurological manifestations within 0.5–1 hour after injection, including clenched fists, latericumbent positioning, opisthotonos and torsion spasms, mimicking the ABE features of human newborns. Meanwhile, no abnormal neurological behavior was observed in the controls, indicating that the CNS manifestation is caused by bilirubin neurotoxicity.

The bodyweight gain on PMD-1 indicated that the feeding behavior of bilirubin-treated rats was influenced by bilirubin neurotoxicity. Additionally, the bodyweight gain of the bilirubin-treated rats on PMD-1 was the lowest, most likely resulting from an extremely severe general condition on the first day; meanwhile, the rats in the bilirubin group showed neurological symptoms on PMD-1. Thus, both the feeding behavior and the manifestations may indicate that PMD-1 represents clinical phase 1 or 2 of ABE [26]–[27]. The feeding difficulty and neurological abnormality partly relieved on PMDs-2/3, showing that the rats' condition improved gradually and indicating that PMDs-2/3 may represent clinical phase 3, similar to the process of kernicterus in human newborns [26]–[27]. The daily bodyweight gain in the bilirubin-treated rats for the first three consecutive days after bilirubin injection indicated that a one-time injection of bilirubin solution could have a persistent impact on the rats' feeding behavior, which most likely could indicate a continuous neurological impact. Moreover, the early mortality rate in the bilirubin group was 19.05%, which is similar to that of moderate ABE in our clinical investigation [5]. Based on the clinical manifestations and the corresponding durations, the bilirubin dosage for a one-time injection could produce a moderate ABE rat model. Therefore, it becomes possible to establish ABE models of different severities (mild, moderate or severe) by adjusting the bilirubin dosage.

According to the American Academy of Pediatrics (AAP), ABE refers to the neurological features of the acute phase, whereas kernicterus is used to describe the chronic and permanent clinical sequelae of bilirubin toxicity, including learning difficulty, motor-development delay, sensorineural hearing loss, epilepsy, cerebral palsy and mental retardation [1]. In this study, the Morris water maze results showed that the bilirubin-treated rats performed significantly worse than the controls, both in the place navigation ability test and the spatial probe trial, indicating that the bilirubin-treated rats had learning and memory deficits at the age of 28 days, comparable to the clinical features in preschool children with kernicterus. Thus, bilirubin injection into the CM resulted in chronic learning and memory disorders in the rats (some also showed unstable gait; data not shown), consistent with the definition of kernicterus in human. Previous studies have shown that some models exhibit chronic sequelae, such as motor delay and ataxia [6]–[11], [14]–[15]. However, to the best of our knowledge, no learning or memory disorders have been confirmed in these models. Therefore, this kernicterus model might be suitable for the study of disorders related to learning and memory which are more and more common among ABE survivors due to the great strides in neonatal care.

Many studies have demonstrated that the basal ganglia, globus pallidus, subthalamic nucleus and hippocampus are typically predominant regions affected by bilirubin; among these, the hippocampus is believed to play an important and essential role in learning and memory [28]–[31]. In this study, H&E staining and TUNEL and FCM assays showed that bilirubin resulted in nerve cell apoptosis and necrosis in the hippocampus, explaining why bilirubin-treated rats performed worse on the Morris water maze test and indicating that this model might better demonstrate the clinical features of kernicterus.

Currently, the known bilirubin neurotoxicity involves multiple mechanisms, including the following: neuronal excitotoxicity, increased intracellular calcium concentration, mitochondrial energy failure, release of excessive NO and impaired long-term synaptic plasticity. These mechanisms trigger downstream events such as the activation of proteolytic enzymes, apoptotic pathways and necrosis [32]–[34]. Many *in vitro* studies have demonstrated that bilirubin induces the apoptosis and necrosis of nerve cells originating from specialized cell lines or primary culture [35]–[38]. Apoptosis and necrosis represent the extremes of a cell death spectrum in which the extent of the insult, bilirubin concentration and exposure time may play a critical role [37]. In this study, the Western blotting results suggested that UCB CM injection could induce hippocampal apoptosis by stimulating cytochrome-c release from the mitochondria to the cytoplasm. Furthermore, the expression of mitochondrial Bax was upregulated, while the expression of mitochondrial Bcl-2 and Bcl-xl was downregulated 24 hours after bilirubin injection. The proapoptotic/antiapoptotic ratio is a crucial factor that determines whether cells undergo apoptosis [39]. The results strongly demonstrated that bilirubin could induce apoptosis in the hippocampus via the mitochondrial pathway *in vivo*. Apoptosis is much more important in neonatal brain damage than in adult brain damage [40], which suggests that inhibiting apoptosis in nerve cells might be an effective strategy for preventing bilirubin-induced brain damage. Furthermore, the FCM assay results indicated that the apoptosis and necrosis of hippocampal cells both occurred *in vivo* after bilirubin injection. Hence, these findings emphasize the need to prevent necrosis as well in order to protect hippocampal nerve cells from bilirubin neurotoxicity.

In summary, there are some advantages to this novel kernicterus SD rat model. First, the model presents not only the characteristic manifestations of ABE after UCB injection but also shows the features of chronic sequelae. Therefore, it might exhibit the entire course of bilirubin encephalopathy [1], [26]–[27]. Second, we injected UCB into the CM to more directly study bilirubin neurotoxicity. Third, as CM injection is easy to perform, the achievement of more stable models is feasible [16]–[19]. Finally, ordinary SD rats can be used instead of Gunn rats, *Ugt1*-null mice or humanized *Ugt-1* mice, thus, the low cost makes this model feasible for most researchers and laboratories, regardless of economic condition.

## Supporting Information

Figure S1 **Bilirubin dose-response curve.** (A) Dose-response curve of the mortality rate within 7 days after bilirubin injection. (B) Dose-response curve of the bodyweight gain on post-modeling days 1-3 (PMDs-1/2/3) after bilirubin injection. (C) Dose-response curve of the scores (a) and the incidence (b) of clinical manifestations within two hours after bilirubin injection. Note: The most common clinical manifestations, including clenched fists, opisthotonos, latericumbent positioning and rolling, were included in the clinical manifestation scores. If the rat showed one of these manifestations within two hours after bilirubin injection, one point was recorded; similarly, if the rat showed four symptom manifestations, four points were recorded.
(TIF)

Figure S2 **The autopsy showed yellow staining of the cerebrum and pons after bilirubin injection.** (A) Bilirubin group; (B) Control group. Coronal section: (a) bregma 0.0 mm, (b-c) bregma −2.0 mm, (d) bregma −7.0 mm. Bilirubin staining (red arrow) and a cavity (black arrow) were observed in the cerebrum of the bilirubin-treated rats (A-a, b, c, d), and the pons was also stained with bilirubin (A-d). Bilirubin staining and cavity formation were not observed in the controls (B-a, b, c, d).
(TIF)

File S1 **The ARRIVE Guidelines Checklist.**
(DOC)

Movie S1 **Bilirubin rat shows opisthotonos and torsion spasms within 0.5–1 hour after bilirubin injection.**
(AVI)
