# Supplementary figures and images for: A Novel Newborn Rat Kernicterus Model Created by Injecting a Bilirubin Solution into the Cisterna Magna
Source: PLoS One. 2014 May 5;9(5):e96171. doi: 10.1371/journal.pone.0096171 (PMC4010446; doi:10.1371/journal.pone.0096171)

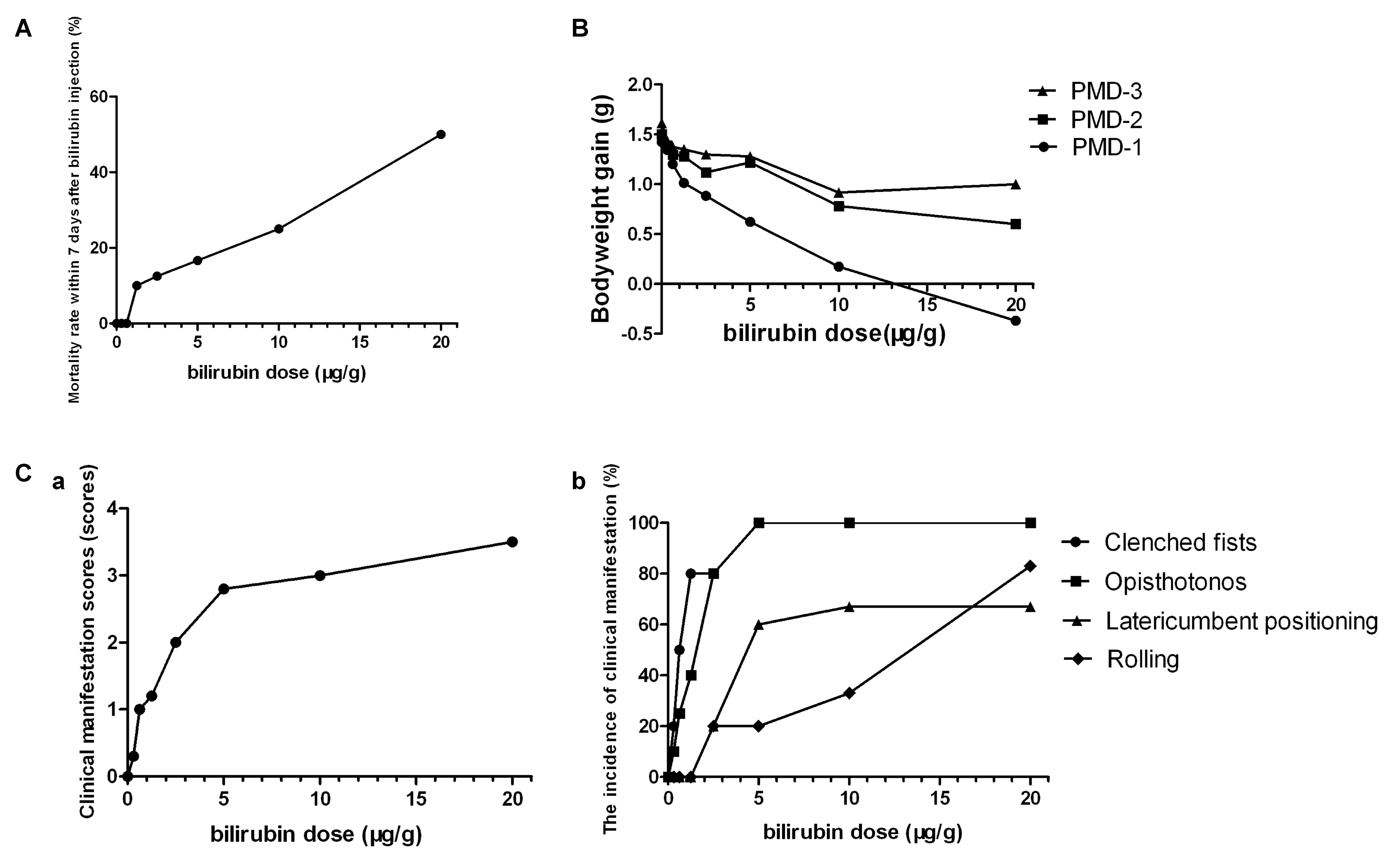

Supplement: Figure S1 — Bilirubin dose-response curve. (A) Dose-response curve of the mortality rate within 7 days after bilirubin injection. (B) Dose-response curve of the bodyweight gain on post-modeling days 1-3 (PMDs-1/2/3) after bilirubin injection. (C) Dose-response curve of the scores (a) and the incidence (b) of clinical manifestations within two hours after bilirubin injection. Note: The most common clinical manifestations, including clenched fists, opisthotonos, latericumbent positioning and rolling, were included in the clinical manifestation scores. If the rat showed one of these manifestations within two hours after bilirubin injection, one point was recorded; similarly, if the rat showed four symptom manifestations, four points were recorded. (TIF) [file pone.0096171.s001.tif]

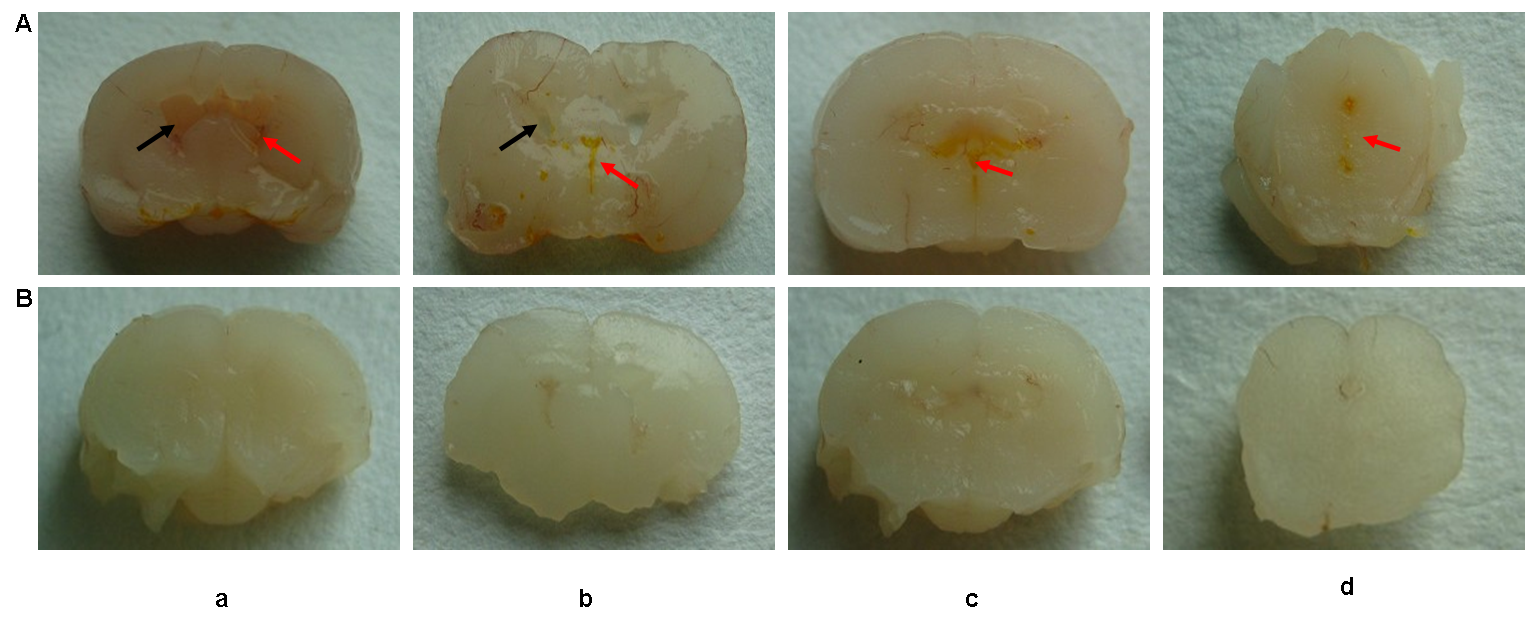

Supplement: Figure S2 — The autopsy showed yellow staining of the cerebrum and pons after bilirubin injection. (A) Bilirubin group; (B) Control group. Coronal section: (a) bregma 0.0 mm, (b-c) bregma −2.0 mm, (d) bregma −7.0 mm. Bilirubin staining (red arrow) and a cavity (black arrow) were observed in the cerebrum of the bilirubin-treated rats (A-a, b, c, d), and the pons was also stained with bilirubin (A-d). Bilirubin staining and cavity formation were not observed in the controls (B-a, b, c, d). (TIF) [file pone.0096171.s002.tif]
